# Outcomes and Risk Factors for Mortality among Patients Treated with Carbapenems for *Klebsiella* spp. Bacteremia

**DOI:** 10.1371/journal.pone.0143845

**Published:** 2015-11-30

**Authors:** Lauren R. Biehle, Jessica M. Cottreau, David J. Thompson, Rachel L. Filipek, J. Nicholas O’Donnell, Todd M. Lasco, Monica V. Mahoney, Elizabeth B. Hirsch

**Affiliations:** 1 University of Houston, Houston, Texas, United States of America; 2 Catholic Health Initiatives St. Luke’s Health Baylor St. Luke’s Medical Center, Houston, Texas, United States of America; 3 Northeastern University, Boston, Massachusetts, United States of America; 4 Beth Israel Deaconess Medical Center, Boston, Massachusetts, United States of America; University of Wisconsin Medical School, UNITED STATES

## Abstract

**Background:**

Extensive dissemination of carbapenemase-producing Enterobacteriaceae has led to increased resistance among *Klebsiella* species. Carbapenems are used as a last resort against resistant pathogens, but carbapenemase production can lead to therapy failure. Identification of risk factors for mortality and assessment of current susceptibility breakpoints are valuable for improving patient outcomes.

**Aim:**

The objective of this study was to evaluate outcomes and risk factors for mortality among patients treated with carbapenems for *Klebsiella* spp. bacteremia.

**Methods:**

Patients hospitalized between 2006 and 2012 with blood cultures positive for *Klebsiella* spp. who received ≥ 48 hours of carbapenem treatment within 72 hours of positive culture were included in this retrospective study. Patient data were retrieved from electronic medical records. Multivariate logistic regression was used to identify risk factors for 30-day hospital mortality.

**Results:**

One hundred seven patients were included. The mean patient age was 61.5 years and the median APACHE II score was 13 ± 6.2. Overall, 30-day hospital mortality was 9.3%. After adjusting for confounding variables, 30-day mortality was associated with baseline APACHE II score (OR, 1.17; 95% CI, 1.01–1.35; *P* = 0.03), length of stay prior to index culture (OR, 1.03; 95% CI, 1.00–1.06; *P* = 0.04), and carbapenem non-susceptible (imipenem or meropenem MIC > 1 mg/L) infection (OR, 9.08; 95% CI, 1.17–70.51; *P* = 0.04).

**Conclusions:**

Baseline severity of illness and length of stay prior to culture were associated with 30-day mortality and should be considered when treating patients with *Klebsiella* bacteremia. These data support the change in carbapenem breakpoints for *Klebsiella* species.

## Introduction

Bloodstream infections (BSI) are one of the four most common hospital acquired infections and a significant cause of morbidity and mortality in hospitalized patients [1]. Over time, the pathogens causing BSI have shifted toward an increase in Gram-negative bacteria as well as an increase in prevalence of antibiotic-resistant strains [2]. These Gram-negative BSI often lead to prolonged hospitalization, higher economic burden, and increased mortality [1]. Carbapenems have been used as a last resort for resistant Gram-negative infections; however, the recent increase in carbapenemase-producing Enterobacteriaceae (i.e. *Klebsiella pneumoniae* carbapenemases [KPC] and New Delhi metallo-beta-lactamases [NDM]) has led to carbapenem failures and suboptimal patient outcomes [3,4,5,6,7,8].

The Clinical and Laboratory Standards Institute (CLSI) and the European Committee on Antimicrobial Susceptibility Testing (EUCAST) have both lowered the susceptibility breakpoints for carbapenems against Enterobacteriaceae to reflect these increases in resistance. Appropriate pharmacotherapy, as identified by these interpretive criteria, contributes to patient outcomes. As resistance mechanisms continue to evolve among Enterobacteriaceae, identification of independent risk factors for mortality may also influence the choice of pharmacotherapy. The objective of this study was to evaluate outcomes and risk factors for 30-day hospital mortality among patients treated with carbapenems for *Klebsiella* spp. bacteremia.

## Materials and Methods

### Ethics statement

All procedures followed were in accordance with the ethical stands of the responsible committee on human experimentation (institutional and national) and with the Helsinki Declaration of 1975, as revised in 2000 and 2008. The study was approved by the institutional review boards of CHI St. Luke’s and Beth Israel Deaconess Medical Center. Due to the retrospective study design, informed consent from subjects was waived

### Study sites

The study was conducted at Catholic Health Initiatives St. Luke’s Health-Baylor St. Luke’s Medical Center (CHI St. Luke’s) in Houston, Texas, USA, and Beth Israel Deaconess Medical Center (BIDMC) in Boston, Massachusetts, USA. CHI St. Luke’s is an 880-bed tertiary care and university-affiliated hospital in the Texas Medical Center. BIDMC is a 649-bed teaching hospital of Harvard Medical School.

### Study design and patients

This retrospective cohort study evaluated adults hospitalized from January 2006 through December 2012 with at least one positive *Klebsiella* spp. blood culture. The study was approved by the institutional review boards of CHI St. Luke’s and BIDMC. Due to the retrospective study design, informed consent from subjects was waived.

All patients, (age ≥ 18 years), with *Klebsiella* spp. BSI during the study period were identified from the clinical microbiological laboratory database and were included if they met the following criteria: minimum of one positive *Klebsiella* spp. blood culture (i.e., index culture); received treatment with a carbapenem (doripenem, ertapenem, imipenem, or meropenem) within 72 hours of the index culture; and received a minimum of 48 hours of carbapenem therapy. Patients were excluded if they had a BSI within the previous 30 days or had a polymicrobial BSI evidenced by positive blood cultures for any organism other than *Klebsiella* spp.

The primary endpoint of the study was 30-day (all-cause) hospital mortality from the date of the index culture. Patients discharged from the hospital within 30 days were deemed to be alive unless proven otherwise.

### Definitions

Patient isolates were considered carbapenem susceptible if the imipenem / meropenem / doripenem MIC was ≤ 1 mg/L or carbapenem non-susceptible if the imipenem / meropenem / doripenem MIC was > 1 mg/L, as determined by automated susceptibility testing [9]. Both study sites used the Vitek 2 (bioMérieux, Durham, North Carolina) for automated testing during the entirety of the study. If > 1 carbapenem was tested on the panel, we considered the isolate non-susceptible if MIC was > 1 mg/L for at least one agent [10]. Meropenem was the formulary carbapenem at CHI St. Luke’s until June 2010 when doripenem became the formulary agent. Both imipenem and meropenem were formulary at BIDMC until 2011 when meropenem became the primary carbapenem; in November 2012, meropenem was unavailable so the substitution to imipenem was made. Empiric carbapenem therapy was considered that started within 24 hours of the index culture; definitive carbapenem therapy was that initiated > 24 hours after the index culture and continued for two days or more, unless otherwise documented in the chart [11]. Active combination therapy was defined as receipt of (not necessarily concomitantly) at least one additional non-carbapenem agent with in vitro activity, for a minimum of 24 hours, within 72 hours of the positive index culture. Source of BSI was determined by either concomitant positive cultures for *Klebsiella* spp. from other anatomical sites, or as documented by providers in chart notes.

### Statistical analysis

Logistic regression was used to explore risk factors for 30-day hospital mortality. Independent variables assessed included age, APACHE II score at time of index culture, hospital length of stay, length of hospital stay prior to index culture, gender, ethnicity, admission status, co-morbidities, bacteremia source, receipt of active combination therapy, and infection with a carbapenem non-susceptible isolate. The dependent variable was 30-day (all-cause) hospital mortality from the date of index culture. Variables found to be significant (*P* < 0.2) in the univariate analyses were included in the multivariate model, where backward selection process was utilized. A *P* value of ≤ 0.05 was considered significant unless stated otherwise. All statistical analyses were performed using Systat® version 13.0 (Systat Software, Inc., San Jose, CA, USA).

## Results

### Patients

A total of 107 unique patients with *Klebsiella* spp. BSI were included; the majority (n = 100) of BSI were caused by *K*. *pneumoniae* while 7 were caused by *K*. *oxytoca* (all in the carbapenem-susceptible group). Clinical characteristics of the patients are presented in Table 1. The mean patient age was 61.5 ± 15.4 years and the mean APACHE II score was 13.8 ± 6.2. The majority of patients (76%) were admitted from home and the median length of stay prior to the index culture was 11.5 days. Patients with carbapenem non-susceptible infections (n = 7) had isolates with MICs ranging from 2 mg/L to > 8 mg/L. Two patients had isolates with an MIC of 2 mg/L (one meropenem; one imipenem), one patient had an isolate with an MIC of 4 mg/L (imipenem), three patients had isolates with an MIC of 8 mg/L (one meropenem; two imipenem), and one patient had an isolate with an MIC ≥ 16 mg/L (listed as “R” [both imipenem and meropenem]).

**Table 1:** Clinical characteristics of the study patients (n = 107).

Characteristic		n = 107
**Age, mean ± SD**		61.5 ± 15.4
**Male, n (%)**		57 (53.3)
**APACHE II, mean ± SD**		13.8 ± 6.2
**Ethnicity, n (%)**		
	Caucasian	61 (57)
	African American	29 (27.4)
	Hispanic	14 (13.1)
	Other	1 (2.8)
**Admit status, n (%)**		
	From home	81 (75.7)
	From OSH	15 (14.3)
	From SNF/LTAC	11 (10.3)
**Length of stay, days**		
	Hospital, mean ± SD	27.8 ± 36
	Hospital, median (range)	14 (1–258)
	Prior to positive culture, mean ± SD	18.4 ± 20.6
	Prior to positive culture, median (range)	11.5 (2–141)
**Co-morbidities** ^**a**^ **, n (%)**		
	Type 2, Diabetes mellitus	39 (36.4)
	Immunosuppression	38 (35.5)
	Hepatic	28 (26.2)
	Renal	26 (24.3)
	Congestive heart failure	25 (23.4)
	Central nervous system	11 (10.3)
	Respiratory	10 (9.3)
**Source of infection** ^**b**^ **, n (%)**		
	Urinary	31 (28.9)
	Intra-abdominal	30 (28)
	Line	13 (12.2)
	Respiratory	4 (3.7)
	Skin/soft tissue	3 (2.8)
	Unknown	32 (29.9)
**Carbapenem therapy, n (%)**		
	Empiric	79 (73.8)
	Definitive	28 (26.2)
**Receipt of active combination therapy** ^**c**^ **, n (%)**		47 (50.5)
**30-day hospital mortality, n (%)**		10 (9.3)

^a^ Co-morbidities included: immunosuppression–organ transplantation, chronic steroid therapy (>10 mg of prednisone or equivalent daily for >1 month), post chemotherapy, human immunodeficiency viral infection; hepatic–hepatitis, cholangitis, cirrhosis; renal–chronic renal insufficiency; central nervous system–stroke, cerebrospinal fluid leak; respiratory–chronic obstructive pulmonary disease, asthma.

^b^ Does not add up to 100% if patient had ≥ 1 source of bacteremia identified

^c^Defined as ≥ 24 hours of therapy with a non-carbapenem with in vitro activity, within 72 hours of index culture. Assessed for n = 93 as some records unavailable.

### Antibiotic consumption

The most common carbapenem utilized was meropenem (n = 64; 60%), followed by ertapenem (n = 30; 28%), doripenem (n = 15; 14%), and imipenem (n = 8; 7%). A total of 10 patients received two carbapenems during their hospital course, generally a reflection of a switch from meropenem to ertapenem (n = 8). The majority of patients received empiric carbapenem therapy (n = 79; 73.8%) while 26.2% received definitive therapy. The median duration of carbapenem therapy was six days (range 2–39 days). Fifty percent (47 / 93 evaluable) of patients received active combination therapy. Of the 47 patients who received active combination therapy, 25 received aminoglycosides (53%), 13 received piperacillin-tazobactam (28%), 9 received an active cephalosporin (19%), and 8 received an active fluoroquinolone (17%).

### Outcomes

Overall, 30-day all-cause hospital mortality was 9.3% (n = 10). Multivariate regression (Table 2) revealed 30-day mortality was associated with APACHE II score at time of index culture (OR, 1.17; 95% CI, 1.01–1.35; *P* = 0.03), length of hospital stay prior to index culture (OR, 1.03; 95% CI, 1.00–1.06; *P* = 0.04), and infection with a carbapenem non-susceptible isolate (OR, 9.08; 95% CI, 1.17–70.51; *P* = 0.04).

**Table 2:** Logistic regression analysis for the risk factors for 30-day hospital mortality. Only variables with *P* < 0.2 in the univariate analysis are shown.

Variables		Univariate analysis; OR (95% CI)	*P* value	Multivariate analysis; OR (95% CI)	*P* value
**Male**		3.92 (0.79–19.40)	0.09		
**APACHE II at index culture**		1.16 (1.04–1.30)	< 0.01	1.17 (1.01–1.35)	0.03
**Hospital length of stay**		1.02 (1.01–1.04)	< 0.01		
**Length of stay prior to culture**		1.03 (1.00–1.06)	0.03	1.03 (1.00–1.06)	0.04
**Co-morbidities** ^**a**^					
	Renal	3.62 (0.96–13.69)	0.06		
	Immunosuppression	3.05 (0.80–11.57)	0.10		
**Source of bacteremia**					
	Intra-abdominal	2.88 (0.77–1.79)	0.12		
**Carbapenem non-susceptible infection**		9.96 (1.85–53.60)	< 0.01	9.08 (1.17–70.51)	0.04

Receiver operating characteristic of the final model = 0.872

^a^ Co-morbidities included: immunosuppression–organ transplantation, chronic steroid therapy (>10 mg of prednisone or equivalent daily for >1 month), post chemotherapy, human immunodeficiency viral infection; renal–chronic renal insufficiency.

## Discussion

The results of our study suggest significantly higher hospital mortality as a result of infections considered carbapenem non-susceptible, increasing baseline APACHE II score, and length of stay prior to culture. Other studies highlighting risk factors associated for mortality in *Klebsiella* spp. BSI have similarly demonstrated increased risk of mortality with increased severity of the patient’s overall condition [12]. A recent meta-analysis of Enterobacteriaceae BSI demonstrated an association of severity of patient condition with independent risk of mortality in seven studies [13]. Five of the studies also demonstrated carbapenem resistance as an independent predictor of death.

A recent observational study of 205 patients with carbapenemase-producing *Klebsiella* BSI also demonstrated an increased mortality rate with increasing carbapenem MIC, however an MIC cutoff of 8 mg/L was used. Mortality rates of patients who received carbapenems (n = 79) were 19.3% (MIC ≤ 8 mg/L) vs. 35.5% (MIC > 8 mg/L). Our study differs because groups were stratified based on a breakpoint of ≤ 1 mg/L, rather than an MIC of ≤ 4 or 8 mg/L [5,13]. Our study is also unique in that we only evaluated patients with monomicrobial *Klebsiella* spp. BSI. Other studies evaluated clinical outcomes in patients with polymicrobial BSI. Since this patient population was excluded from our study, the influence of the virulence of other organisms was unlikely to be a contributing factor [5,14,15] and the mortality risk factors identified in our multivariate regression were likely less confounded by infection with other species.

In carbapenemase-producing *Klebsiella* BSI, combination therapy has previously been demonstrated to be associated with survival [5,7,8]. In our evaluation, combination therapy was not associated with a survival effect, but only a minority of patients had carbapenem non-susceptible infections. Definitions of combination therapy may vary and our definition of combination active therapy was liberal as a minimum of 24 hours of therapy with the other active agent(s); thus, patients may not necessarily have received true concomitant therapy with a second active agent, but the additional agent may have contributed to patient outcome so it could not be ignored.

In 2010, the Clinical and Laboratory Standards Institute (CLSI) lowered the minimum inhibitory concentration (MIC) susceptibility breakpoints for carbapenems (i.e., imipenem, meropenem, and doripenem at standard doses) against Enterobacteriaceae from 4 mg/L to 1 mg/L. MIC susceptibility breakpoints for ertapenem have also been modified, with the most recent change to 0.5 mg/L. The rationale for this update included updated pharmacodynamic and pharmacokinetic data, as well as the increased isolation of KPC throughout the United States. Current breakpoints by the European Committee on Antimicrobial Susceptibility Testing, though higher than CLSI, also reflect increasing resistance with imipenem and meropenem breakpoints of 2 mg/L and doripenem of 1 mg/L. (http://www.eucast.org/clinical_breakpoints/) Among patients receiving carbapenem therapy, our evaluation supports the lowered breakpoints for carbapenems as infections with an MIC > 1 mg/L were found to be independently associated with mortality.

Our study is not without limitations. As a small, retrospective, observational study, we were unable to control for prescribing practice of antimicrobials. As a result, there was heterogeneity in the carbapenem selected for treatment and time to initiation of therapy by providers. Patients with carbapenem non-susceptible infection were limited; however, the majority of isolates displayed low-level carbapenem resistance (MICs ranged from 2 to 8 mg/L), categorizing them as susceptible or intermediate per the pre-2010 CLSI breakpoints. Lastly, only one of the carbapenem non-susceptible isolates was available for genotypic analysis and was confirmed as a KPC producer [16,17]. Additional, previous investigations at CHI St. Luke’s have shown that KPC production is a leading mechanism mediating carbapenem resistance [17].

In summary, this study suggests significantly higher mortality in carbapenem-treated patients with increased APACHE II scores, length of stay prior to culture, and in BSI caused by isolates with an MIC > 1 mg/L, supporting the change in carbapenem breakpoints for Enterobacteriaceae. As resistance rates continue to increase among Enterobacteriaceae, identifying optimal regimens for carbapenem non-susceptible infections and the use of appropriate initial therapy will play an important role in improving outcomes for patients with BSI.
